# Family Members’ Experiences of Long-Term Home Care for Older Adults Provided by Live-In Migrant Caregivers: A Meta-Synthesis of Qualitative Studies

**DOI:** 10.3390/healthcare14040483

**Published:** 2026-02-13

**Authors:** Sandra Aliaga-Castellanos, Sergio Martínez-Granero, Alba Fernández-Férez, José Granero-Molina, Laura Helena Antequera-Raynal, Gonzalo Granero-Heredia, María del Mar Jiménez-Lasserrotte

**Affiliations:** 1 Nursing, Physiotherapy and Medicine Department, University of Almeria, 04120 Almería, Spain; salicaste@gmail.com (S.A.-C.); smg198@inlumine.ual.es (S.M.-G.); lar855@ual.es (L.H.A.-R.); ggh736@inlumine.ual.es (G.G.-H.); mjl095@ual.es (M.d.M.J.-L.); 2 Distrito Sanitario Poniente, 04750 Almería, Spain; alba.fernandez.ferez.sspa@juntadeandalucia.es; 3 Facultad de Ciencias de la Salud, Universidad Autónoma de Chile, Santiago 7500000, Chile; 4 Hospital Vithas, 04120 Almería, Spain

**Keywords:** long-term home care, migrant caregivers, family caregivers, older adult, qualitative research

## Abstract

**Background/Objectives:** The aim of this study was to synthesise qualitative evidence from family members’ experiences of long-term home care for older adults provided by live-in migrant caregivers. **Methods:** We conducted a systematic literature review with meta-synthesis using four online databases. The search included articles published between January 2016 and December 2025 on the CINAHL, PubMed, SCOPUS and WOS databases. Thematic synthesis of qualitative data was conducted. **Results:** Eleven papers from six different countries fulfilled the criteria and were included in the thematic synthesis. Four main themes were identified: 1. Not an easy decision. 2. A stranger at the heart of family life. 3. Two worlds that meet and need each other. 4. Improving the integration of migrant caregivers into family life. Hiring migrant caregivers to provide long-term home care for older adults can ease the burden on family caregivers, but it is an additional source of stress and worry. **Conclusions:** The family members of older adults call for greater financial and institutional support, as well as the involvement of social and health services in the training and education of families and migrant caregivers. Negotiation skills and the ability to reach consensus between older adults (OAs), family members and resident migrant caregivers are key to improving cohabitation and care for OAs. The primary goal is the well-being of the OAs, which involves overcoming cultural prejudices, learning together in response to the new situation, improving caregivers’ training, and ensuring continuity of care.

## 1. Introduction

The ageing process is changing in modern societies; global projections indicate that 16% of the population will be over 65 years old by 2050 [1]. The increase in scientific research, technical and social advances, and improved hygiene, sanitation and public health have led to increased life expectancy in developed countries [2]. Spain leads the European Union in population ageing, with 20.1% of the population over the age of 65 and a life expectancy of 83.07 years [3]. This demographic challenge poses a threat to welfare models as it increases multimorbidity, chronicity [4], frailty [5], dependency [6], loneliness and social isolation [7] among older adults (OAs). Furthermore, changes in family structure [8] call for innovative care strategies with long-term care (LTC) systems [9]. The WHO defines LTC systems as “national systems that ensure integrated long-term care that is appropriate, affordable, accessible and upholds the rights of older people and carers alike” [10]. LTC includes management of chronic geriatric conditions, rehabilitation, palliative care, health promotion [11], basic care, and social support for OAs [12]. Increased life expectancy increases the long-term care needs of OAs [13,14], which are attended to by formal and informal carers (family members, friends or neighbours) [15]. Traditionally, mothers, wives, and daughters have cared for OAs in the home [16], but the entry of women into the labour market and the fact that most adult children do not live with their parents [17] has increased the demand for home caregivers [18,19]. Some countries have adopted measures to enable OAs to remain in their homes for as long as possible, such as home adaptations, integrated care, and the promotion of informal and/or migrant caregivers [20]. Many family members are forced to reduce their working hours to provide care for OAs, putting themselves at risk of economic hardship [21,22]. Many OAs prefer to be cared for at home, but the shortage of institutional LTC and high costs lead families to hire migrant caregivers [16,17,18,19]. Hiring migrant caregivers is a strategy to address LTC needs in OAs [23]. Family members hire live-in migrant caregivers so that OAs can remain in their own homes, and to reduce the workload of informal caregivers [24]. Twenty-four-hour care for OAs is thus transferred to live-in migrant caregivers, who live in the household and provide paid services [25]. The hiring of live-in migrant caregivers varies across European countries, taking place either through agencies or via direct arrangements between older adults and/or their families [20]. Europe is a preferred destination for migration [26]; Spain has 6.1 million immigrants, half of whom are women [3]. In 2022, 80.7% of migrant women worldwide were employed in services, mainly care and domestic work [27]. In the same year, there were 375,000 people in Spain registered in the Special System for Domestic Workers, of whom 165,000 were migrants and 94% were women [28]. OAs, family members and live-in migrants constitute a ‘home care triad’ [29]. Informal caregivers tend to be migrant women [30]. Hiring a migrant for LTC to OAs involves changes in family dynamics/roles, loss of privacy, and concerns about training and safety [19,31]. Several studies have explored the experiences of OAs and migrant caregivers in the home [32,33,34,35,36,37], but there is little evidence of family caregiver experiences [38]. Our research question is: what are the experiences of family members regarding the care of OAs by live-in migrant caregivers? The objective of this study was to synthesise qualitative evidence from family members’ experiences of long-term home care for OAs provided by live-in migrant caregivers.

## 2. Materials and Methods

### 2.1. Design

This is a systematic review and meta-synthesis of qualitative studies [39]. This review follows the PRISMA 2020 statement guideline for reporting systematic reviews, as well as the ENTREQ (Enhancing Transparency in Reporting the Synthesis of Qualitative Research) guidelines (Supplementary Materials) [40].

### 2.2. Search Methods

An advanced bibliographic search for qualitative studies in English and Spanish published between January 2016 and December 2025 was carried out in the following Health Sciences databases: CINAHL, PubMed, Scopus and Web of Science. The SPIDER method was used to formulate the research question: Sample (family caregivers, relatives), Phenomenon of Interest (LTC of OAs by live-in migrants); Design (qualitative descriptive study, phenomenological study, etc.); Evaluation (experiences, perceptions, opinions); Research (qualitative research, mixed methods) [41]. The search strategy can be found in Table 1.

### 2.3. Inclusion and Exclusion Criteria

Inclusion criteria were as follows: primary qualitative or mixed-methodology studies, with text preferably in English or Spanish, that include experiences of family members with migrant caregivers who provide LTC to OAs at home. Exclusion criteria were as follows: non-primary articles, editorials, opinion pieces or abstracts; articles on the experiences of family members of OAs cared for by migrants in social and healthcare institutions.

### 2.4. Search Results

**Figure 1:**
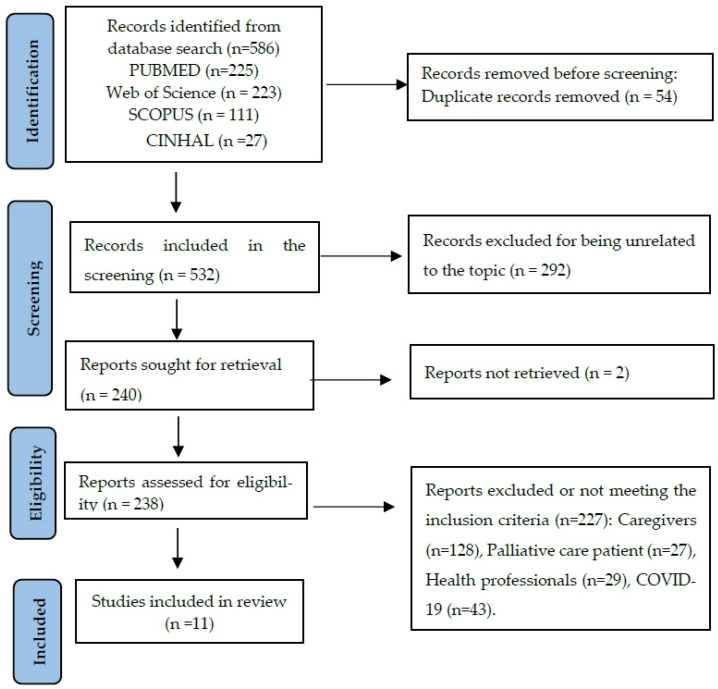
Flowchart.

A 5-stage selection process was performed by three researchers (SMG, GGH and AFF). A total of 772 studies were identified; however, only eleven studies were included in this review (Figure 1).

### 2.5. Data Extraction

Two authors (SAC, LHAR) independently performed data extraction and reached a consensus. Disputes were discussed with a third researcher (JGM). Articles selected in each phase were approved by ≥2/3 of the researchers (Figure 1).

### 2.6. Quality Assessment

Each primary study was assessed using the Joanna Briggs Institute’s Qualitative As-sessment Rating Instrument (QARI) [42,43] (Table 2). The quality assessment was carried out by (SMG, GGH and RFG) and reviewed by (JGM and MMJL). The methodological weaknesses detected were mainly related to the descriptions of the researcher’s cultural and theoretical context [16,31,44], as well as lack of reflexivity or the influence of the researcher on the research [16,18,20,25,31,45,46,47]. Although these aspects could be improved, their influence on the emergent themes of this review is limited. Across the included studies, there is consistent agreement that it is the ‘relational fit’ among those involved which enables families to feel cared for, safe, and relieved, thereby experiencing stability. Disagreements were discussed in online sessions until a consensus was reached. This review included all studies that met ≥70% of QARI quality criteria; no studies were excluded after quality assessment.

### 2.7. Data Synthesis and Analysis

The included studies were analysed thematically and inductively. The synthesis was undertaken by (SAC) and verified by (JGM). Two independent reviewers with expertise in LTC and qualitative research verified the results. The thematic synthesis of qualitative data (Table 3) included line-by-line coding, developing descriptive themes and generating analytical themes in three stages [48]. No computer software was used.

### 2.8. Rigour

To assess validity, structured summaries of each original study were produced. We also verified whether the findings were transferable to other LTC contexts. The authors distinguished between LTC in OAs and other patients with chronic or disabling conditions. In addition, we analysed whether the findings of our synthesis could be attributed to a particular group of migrant caregivers. After the thematic synthesis, we examined the contributions of the studies to the final analytical themes.

## 3. Results

This review includes 11 articles from six countries, with a total of 271 family members of OAs who have a live-in migrant caregiver. The synthesis brought together themes and sub-themes that reflect the experiences of family members of OAs receiving LTC from migrant caregivers. The characteristics of the selected studies can be found in Table 4.

The thematic analysis of the studies led to the identification of four themes and thirteen sub-themes, which can be seen in Table 5 alongside the units of meaning.

### 3.1. Not an Easy Decision

The decision to hire a migrant domestic caregiver stems from the need to care for OAs and the family’s inability to meet these needs. OAs and their families seek 24 h home care when their health condition worsens.

#### 3.1.1. Hiring a Live-In Migrant Caregiver: When Choice Becomes a Necessity

Family members cited various reasons for hiring a migrant caregiver. The main factor was the continuous and progressive deterioration of the health of the OAs, and the impossibility to care for them. This is primarily due to the entry of women into the labour market and is compounded by the effort and burden of providing care:

*“I am the only child, I am working and have three children (…). I was … overwhelmed, I went from home to work, from work to my mother’s home, (…) I stayed with her all night, returned to my home in the morning half asleep, then to work, and again run to cook something to eat”* 

[19].

Living in a family environment is highly valued by OAs and their informal caregivers. Many do not wish to enter a nursing home or to remain alone at home. As one OA expressed:

*“Home is incredibly important for us. When we built our house, we said ‘this is our senior apartment’. This is where we want to grow old”* 

[20].

Sometimes, the decision to hire a home caregiver is made after a critical incident that affects the OA’s health.

*“Why do I hire migrant care workers? Because I work every day, and her (my mother’s) blood sugar is not well controlled”* 

[46].

In general, the decision to hire a caregiver in some families was accelerated by the OA being diagnosed with a highly disabling chronic condition. Families seek help and often find home care services that are provided by different caregivers, with fragmented schedules, and uncovered hours. Faced with this situation, they consider either admitting the OA to a nursing home or hiring a live-in caregiver. Families prioritise that OAs are accompanied, well cared for, and able to maintain a degree of control over their lives.

*“For example, my father had to go to bed at 7 p.m., because that was the time the nurse could be there. So, in fact, their whole life was arranged around the professional home care and not the other way around” (son)* 

[20].

Other times, OAs are admitted to a nursing home, but do not have a positive experience. As one family member says, they do not always adapt well:

*“I would say the main reason was that she wasn’t happy in the nursing homes. (…) she didn’t feel comfortable either” (daughter)* 

[25].

Thus, family members may feel a duty and desire to care for the OA themselves and even try to adapt their lives to do so. However, they often find themselves physically and mentally unable to do so. At this point, tensions arise and hiring a live-in caregiver goes from being a choice to a necessity.

*“We took turns, one months, two months, three months like that. Towards the end we were really tired and we started to point fingers at each other, like: Why do I have to take so much responsibility?”* 

[45].

Another motivation for hiring home caregivers was to reduce the workload of the informal caregiver. The daughter of a care-dependent couple clarified this as follows:

*“So that the people can be taken care of at home and that the family can be at ease. The family can live in a different way. We were always anxious before: ‘I hope they didn’t fall; I hope nothing bad happened”* 

[20].

#### 3.1.2. Demands of the Family Members

Many migrant caregivers spend 24 h a day in the home with the OAs, and their jobs involve a range of household tasks, such as cooking, cleaning, etc. Family members are reluctant to hire young, inexperienced carers, claiming that they need to be taught what to do and do not pay attention to the needs of the OAs. As expressed by the daughter of an OA:

*“Of course they have to be able to deal well with older people with illnesses, have empathy, do a bit of housekeeping, be friendly to the older people, this is very important, and reassuring for the family”* 

[20].

All of the families had certain expectations of their employees when it came to caring for their loved ones, such as treating the OAs with kindness and respect. For some, flexibility was important; they wanted caregivers to be willing to accept changes in working conditions, primarily in terms of hours:

*“She has her free days, but if anything arises, we need her to be available”* 

[19].

Due to the pressure to hire someone quickly, many family members accepted that the caregiver had no training in the field. They assumed that they would know how to take care of OAs based on their own personal experiences. The families often seek to hire middle-aged caregivers with experience in caregiving in their countries of origin. However, they often do not do their job well, and the families must always keep a close eye on them. This is how one family member expressed it:

*“One time my mother was not taken to the toilet in time, so she peed in the bed. That is a sign that [the live-in carer] does not really live up to the expectations… So I will continue to go there all the time to check up”* 

[45].

When family members observed that migrant caregivers did not have sufficient skills to care for the OAs, they tried to teach them. While they valued their initiative and interest to learn, one family member stated that this was not enough, as it was also important for the OAs to accept them, which was not always easy.

*“Oh, that process was painful (…). My mother criticised the live-in carer, saying that she was ugly, that she had an ugly smile… So, it was difficult for her to take care of my mom, since my mom was criticising and insulting her all the time”* 

[36].

Occasionally it is possible to find the right match; the migrant caregiver is very capable and affectionate towards the OA. In such cases, the professional relationship begins to become personal.

*“My mother matters to her. She [the MCW] treats her like a person, not necessarily as someone for whom she works. [It is] more than that”* 

[18].

#### 3.1.3. Becoming an Employer

When hiring a migrant caregiver, families face issues that were previously unfamiliar to them, such as how to apply for financial support, whether the caregiver’s documentation is in order, and whether they have the necessary residence and work permits in the country. Many family members experienced financial difficulties as a result of hiring a caregiver. Having a caregiver in the home 24 h a day increases household expenses for food, water, hygiene products, etc. The family members try to address this issue by assigning domestic tasks to migrant caregivers, for which they have often not been hired.

*“Therefore, in the morning, when (the live-in carer) wakes up, she will mop the floor and do the laundry. And, then, at seven o’clock, when he (the husband) wakes up, she will make milk for him, and give him milk and bread”* 

[36].

This situation is exacerbated if the caregiver asks for a pay rise. Migrant caregivers are interdependent, so improved working conditions for one can lead to demands for the same from others. At first, they accept any conditions, but as time goes by, they ask for better pay and working conditions. As one family member explained, they know that they are needed and take advantage of that.

*“She came to me and said that all her friends got higher salaries… I didn’t have a choice, did I? She would have left if I hadn’t given her more money”* 

[31].

Sometimes, the high demands of caregivers can exceed the financial means of the families. In these cases, the family may consider changing caregivers, which creates uncertainty and which can have devastating consequences.

*“I see her as my family, she is like my sister and my friend… but what happens if she leaves? I don’t know”* 

[45].

As employers, family members found it difficult to draw the line between the needs and whims of migrant caregivers. When items are purchased to meet the needs of the OA, the family or the migrant caregiver, it is difficult to define what ‘basic needs’ are. As one family member expressed her concern:

*“Am I, as her employer, supposed to purchase her cosmetics, such as body lotion and face lotion?”* 

[31].

On other occasions family members worry about the difficult working life of migrant caregivers, the lack of free time, and how they cope:

*“She had to be available for 7 days, 24 h to take care of my father. I thought it was almost slavery. But at the same time, it has been experienced by Linda (live-in carer) as an improvement in her situation” (son)* 

[20].

It is difficult for family members to act as mediators between caregivers and OAs, especially if they have to defend the caregiver or negotiate to avoid conflict. OAs may present cognitive or behavioural problems, delusions, obsessions, etc., and in the event of a difference of opinion, it is difficult to know who is telling the truth.

### 3.2. A Stranger at the Heart of Family Life

When hiring a domestic caregiver, the family members are not merely employers; a personal relationship is established between the OA, the family, and the caregiver. Initially, there is a sense of unfamiliarity and mistrust, which gradually gives way to a relationship of interdependence and trust.

*“You don’t really know what kind of person is coming and how it will go and how they will react. (…) It’s a bit of an adventure” (daughter)* 

[25].

#### 3.2.1. Mixed Feelings: Between Doubt and Trust

It was clear that trust had been established when the family members started to give the caregivers more responsibilities, supervised them less, helped them with their work, and treated them like part of the family. This trust was reinforced when the caregiver had a good attitude, treated the OA well, and was decisive. Nonetheless, some participants still felt uneasy about not knowing what happened at home or how the migrant caregiver treated their family member in their absence.

*“They need to be watched closely because, at the first opportunity they get, they will care only for themselves”* 

[31].

This situation led some family members to install 24 h surveillance cameras in their homes:

*“Of course, a lot of live-in carers are in families where they put up monitors or cameras and they are afraid that the live-in carers come only for money, and without any love… [I told the live-in carer] I trust that you will take good care of my sister, and you treat her as your own mother”* 

[45].

Another source of mistrust was related to money. Some family members suspected that caregivers were asking them for more money than they needed and keeping the surplus for themselves.

*“We gave her money to buy groceries, and she said it cost 700 shekels per week, but we realized it was too much for two women, mainly as one of them hardly eats”* 

[31].

This situation is usually resolved when the family member does the shopping for the person receiving care. In other cases, when the relationship between the caregiver and the family member is long-standing, trust is established and everything improves. After a few weeks, the OAs find a balance in the relationship. They even see the live-in caregivers almost as family members, celebrate their birthdays, or give them Christmas gifts.

*“She (migrant home care worker) knows where the money is. I told her, ‘You don’t need to show me,’ I trust you 100%” (spouse)* 

[47].

At this point, the family feels reassured, can balance their personal lives, and can regain their autonomy and personal equilibrium.

*“I feel that I can now be the master of my time management, because in the past, without the help of migrant care worker, the conditions of my parents were sometimes unstable, which made it difficult for me to engage in any activities”* 

[46].

#### 3.2.2. We Are All Interdependent: Grateful but Vigilant

The family members interviewed acknowledged that they depended on the caregivers; without them, it would be very difficult to balance their personal and professional lives with caring for the OA. Most family members have jobs away from home, so having someone to watch over and care for the OA 24 h a day gives them peace of mind.

*“It gives me a lot of comfort. So I don’t need to worry about what is happening at home… I think that the most important thing is that [live-in carer] takes good care of my mother, and then she will be in my family”* 

[45].

Informal caregivers report that migrant caregivers eased their workload. The migrant caregiver provided them with the emotional capacity to personally care for OAs during their days off, without needing to use respite services offered by long-term care programmes:

*“This is a time for me to be alone with her, but there is someone (the migrant care worker) helping me. I feel an extreme need for this kind of service and assistance”* 

[46].

Many family members were highly grateful and tried their best to take care of their domestic workers so that they wouldn’t leave. Nonetheless, relying on a caregiver was a major source of stress. As one family member stated, even if problems were to arise, they wouldn’t dismiss the migrant caregiver because they wouldn’t be able to find another one, and because the care recipient had developed an attachment to them.

*“I’m so dependent on her. She’s my oxygen, and therefore I’m very protective of her. I buy her presents and send gifts to her children… so she will stay with us”* 

[45].

Family members also help migrant caregivers by paying for some of their necessary expenses, and helping them to resolve legal issues or financial burdens that they carry from their countries of origin. There are cases where family members assume responsibility for the migrant caregiver’s health problems:

*“She is a good helper and now she is sick, then I have to be her caregiver right, I mean that’s only fair. So, I told her, ‘Ok, I will find a surgeon here in Singapore to do it and I will settle my mom’ […]”* 

[16].

Over time, both parties realise that they depend on each other.

*“Therefore, no matter what, honesty is crucial (…) truth, transparency, and regular updates” (son)* 

[47].

However, there were also family members who did not develop attachment to the caregiver because of their lack of empathy, mistreatment of the OAs, or failure to comply with working conditions.

*“It’s the same way as when I go to work, this is her job. She isn’t my friend and she isn’t my family”* 

[18].

### 3.3. Two Worlds That Meet and Need Each Other

Hiring a migrant caregiver can result in positive or negative changes in family dynamics. Even though they provide support, companionship and care to the OAs, while relieving the family member of some responsibilities, problems can also arise due to failure to fulfil their duties or as a result of cultural differences.

#### 3.3.1. A Joint Effort

Although there is initial reluctance, once this phase is overcome, trust is established. Migrant caregivers and family members then join forces to improve the well-being of the OA.

*“As she has a lot of experience already, in the beginning, I just observed a little bit on the side how she interacted with my mom, and then, after a couple of days, I just let her do her stuff. Also, I observed that the energy between the live-in carer and my mom was good”* 

[36].

Furthermore, the migrant caregivers provide the family members with emotional support, help them better understand the OA, and listen to their concerns. Many OAs express their feelings and concerns to the migrant caregivers before talking to their own family members.

*“But my helper is good because she said that if your father knows that he has dementia, he will not behave in this manner. It’s because he doesn’t know, this is dementia she said. And she’s the one who taught us how to manage our temper”* 

[16].

Migrant caregivers carry out or collaborate in tasks to meet the daily needs of the OAs, such as bathing, dressing, and feeding them. As a relative of a person with dementia (PWD) expressed, the support is greater if the migrant caregiver has training:

*“Transferring is a difficult thing because she’s (PWD) totally on us. Because she cannot control herself, and her neck and everything. Luckily my helper was very, very well trained that she can lift her onto the bed independently”* 

[16].

In some cases, family members and migrant caregivers work together or organise shifts. Each family reaches an agreement with the caregiver based on their financial means, availability and work schedule. In addition, family members must cover the caregiver’s rest periods, so that they do not ‘burn out’.

*“Then for the rest of the week, the FDW has to do the day duty, and I do the night duty. We do rotation, otherwise it would be very difficult, very tiring”* 

[44].

The adjustment between migrant caregivers and families takes time and a joint effort to understand each other. As one family member says, this is a recurring, daily problem:

*“Originally, the migrant care worker was supposed to come to solve a problem, but there are also new adaptation issues that need to be addressed”* 

[46].

#### 3.3.2. When Problems Arise

Family members report challenges such as resistance from OAs to welcoming migrant caregivers into their homes, communication barriers, and lack of training, which then leads to tensions:

*“(her mother)… was dissatisfied with her migrant care worker’s behavior, perceiving her as inattentive and wasteful, particularly critical of her use of a mobile phone and arguing back”* 

[46].

Some family members experienced difficulties with the migrant domestic caregivers due to financial issues, deception, or poor attitude. There were instances in which the family members felt manipulated, as caregivers attempted to blackmail them with demands to obtain more money (e.g., sending extra money home, meeting the needs of family members in their country of origin, etc.). The family members’ reliance on the foreign caregivers can cause problems as they become overly dependent and unable to care for the OAs in the caregiver’s absence. Moreover, finding replacements for days off or holidays is very difficult.

*“It was quite challenging because my helper has the day-off, sometimes once a month or twice a month. So, when she is off, we don’t cook. Yah…otherwise, I would have to take leave or I would have to find respite care for my mum”* 

[19].

When migrant caregivers realise that they are essential to family life, they may take advantage of these circumstances and try to extort money from the family by threatening to leave their job. Moreover, the migrant caregivers are a close-knit group who talk to each other and are able to create parallel networks or illegal payment schemes. This is how one family member recounted their experience:

*“We asked Ann to arrange a replacement for her weekends off… She told us how much it cost, and we paid her. But when we spoke with her replacement, we found out that Ann was charging us a commission. This hurt us”* 

[31].

Some family members become emotionally attached to the caregivers, coming to regard them almost as friends or members of the family. However, it should not be forgotten that the caregivers are employees with their own interests. Uncertainty about the retention of migrant caregivers is a constant concern for families. Family members also mentioned irresponsible, unprofessional, or poorly trained migrant caregivers. In these cases, the responsibility for training them falls on the family.

*“I don’t want to change caregivers… because then I would have to train another one and go through the process of adjusting all over again… It’s something that requires working through… I might consider a long-term care [facility] instead”* 

[46].

#### 3.3.3. Culture Shock

Migrant caregivers come from different cultures and enter a home unknown to them. When migrant caregivers were brought into the home, family members and OAs had to go through a process of cultural adaptation. Some migrant caregivers were unfamiliar with the language, beliefs, and customs of the host country, which can lead to problems in the home. It is common for foreign domestic workers with religious beliefs to lean on their religion as a means of coping with adversity. Family members are therefore usually very respectful of the migrant caregiver’s faith. This was expressed by one family member:

*“I don’t stop her practicing her religion… we pray because we are Christians, and we pray for her, and when we come to pray [for my sister-in-law], we don’t force her to pray with us, but sometimes she comes to pray with us”* 

[45].

Bringing a migrant caregiver into the home causes a language barrier that hinders communication with the family. The problem is greater with OAs who have difficulties with hearing, attention or comprehension. According to the family members, this is a fundamental limitation.

*“[She should] learn the language better. She has been here for more than five years… She knows a lot of words in Hebrew and knows a little reading, but to construct a proper sentence—no!”* 

[18].

Some of the OAs showed hostility towards the migrant caregivers, and family members had to negotiate with them to gain their trust. OAs may have travelled less and have fewer relationships with other people, races and cultures.

*“I told my mother we have to get to know her and give her a chance because people cannot be judged by the color of their skin”* 

[19].

The cultural shock is notable as the migrant caregivers have a different way of expressing themselves, as well as different customs regarding food, household hygiene, schedules, and entertainment. In addition, they may feel overwhelmed by their work situation and need to talk about their problems with other caregivers. Family members seem to be understanding of this situation and did not express any issues with the matter.

### 3.4. Improving the Integration of Live-In Migrant Caregivers into Family Life

This section highlights areas for improvement in the care of older adults by migrant caregivers, from the perspective of family members and/or informal caregivers.

#### 3.4.1. Reaching Consensus

In cases of dependency that limit the autonomy of OAs in daily activities, agreements with the family are necessary. It is important to explain the need for caregivers, address the costs, and discuss the advantages and disadvantages of institutional care for OAs versus informal or migrant caregiver support. One son described it as follows:

*“Yes, well, all of us siblings (…) got together. We had a meeting, and I had written down some important questions beforehand (…). And it was simply a question of what kind of help he needed (…). And my siblings then agreed because they saw that we (…) would organize it”* 

[25].

OAs, family members, and migrant caregivers must engage in joint negotiations. This involves considering the opinions of OAs, relinquishing household tasks, building trust with migrant caregivers, and negotiating purchases, meals, living spaces, rest, and other matters.

*“It was also important to try letting my aunt decide where she still has the ability to make decisions, like ‘What are we going to cook, what are we going to buy, where are we going to go now?’ (…) That we don’t just manage her. (…) That someone simply takes the reins and says: ‘Okay, I’m going to do it now.’ Or: ‘You will be cared for now”* 

[25].

#### 3.4.2. Overcoming Cultural Prejudices

The initial period is a process of shared learning, during which OAs, family members, and migrant caregivers gradually get to know one another. A true sense of community only emerges when all parties gain trust and learn to adapt:

*“The openness and willingness to learn of the care migrant also play an important role, as a niece and her aunt explained”* 

[25].

It is difficult to find local caregivers willing to do this type of work. There are companies that offer the service, but many families cannot afford it. Therefore, some family members decided to hire migrant caregivers based on recommendations from acquaintances. However, not all experiences are positive; there are family members who feel reluctant to hire migrant carers due to negative comments from those around them.

*“Whenever we went to the hospital for the list, they recommended, ’no Colombians’, because, in the end, we all know each other”* 

[19].

The absence of a shared cultural perspective can undermine a good caregiving relationship; there is concern about difficulties in understanding and being understood. As one daughter explains:

*“She (migrant home care worker) is in a different place culturally. She doesn’t feel the connection that I do, she doesn’t have the love that I do, she doesn’t provide the care that I do (…) when we bring a human being and expect them to adapt to us, it doesn’t work that way. They come with their ‘bag’, with their life, with their culture, with their character, and they act here according to that, not according to the situation of our parents. Definitely not” (daughter)* 

[47].

#### 3.4.3. Learning Together

OAs, family members, and caregivers must learn to live together, overcoming language barriers and age differences. Families often feel responsible for the well-being of the migrant caregiver, taking an interest in their workload, free time, rest, and salary. They also try to improve the caregiver’s living conditions by providing internet access, a comfortable room, bathroom privacy, ease of transportation, leisure time, and opportunities for social contact:

*“… almost like family members” (son), “almost like a sister to me” (niece), or even “like a daughter” (husband receiving care)* 

[25].

While most family members are satisfied with the quality of care, some have experienced a lack of training among migrant caregivers. Lacking formal instruction, family members took on the role of teaching them how to perform certain tasks, as one couple explained:

*“He has no medical knowledge. That in itself is the problem. I have trained him as far as I could. But the initiative has to come from him and there’s not much there”* 

[20].

For example, when caring for OAs with dementia, caregivers may be competent in basic tasks but not necessarily fostering independence. This is how one family member expressed their concern:

*“My mother gradually became reliant… [then later] too reliant, which led to a regression in her habits and abilities”* 

[46].

Families are committed to educating migrant caregivers in the host society, doing everything possible to integrate them into social and family life:

*“We can relieve her so she can get some fresh air, go out to recharge her batteries. I try to come once a week. My sister also tries to visit. We really make an effort and tell her: ‘Go out, take some time for yourself.’ We are interested in her well-being, (…) we need to give her a good feeling, show her that she is important, that we appreciate her work” (daughter)* 

[47].

#### 3.4.4. Ensuring Continuity of Care

Continuity in the caregiver’s services promotes stability in both care and family life. OAs feel accompanied, family members feel secure and at ease in their daily lives, and the migrant caregiver becomes integrated into the family’s network of relationships, restoring peace to the household. As one daughter expressed:

*“Right now, it’s going very well because it’s always the same two women who come. They’ve been alternating for two years. It’s working really well now; when one arrives and the other leaves, we hardly notice it anymore”* 

[25].

As one daughter says, continuity of care is key for elderly people with dementia, who have difficulty adapting to unfamiliar people in their homes:

*“The elderly may exhibit resistance due to this unfamiliarity”* 

[46].

#### 3.4.5. The Well-Being of OAs

Families are pleased not to have to organise the care themselves, and OAs are happy to remain at home with 24 h support. Over time, a meaningful interaction develops between the OA and the migrant caregiver, which becomes the family’s greatest source of satisfaction:

*“Yesterday, I took my mother out, and the migrant care worker accompanied us to the clinic to pick up medication. I even saw my mother in the examination room telling the caregiver, ‘I am very grateful that you are here to take care of me.’ Witnessing this moment was quite comforting for me”* 

[46].

## 4. Discussion

The objective of this study was to synthesise qualitative evidence from family members’ experiences of long-term home care for OAs provided by live-in migrant caregivers. Although hiring a caregiver supports and facilitates care for OAs at home, studies agree that it is not easy for the family [19,31]. The process of hiring a migrant domestic caregiver is difficult as it has a significant social and cultural impact. Recent studies highlight the complexity of care intertwined with the relationships between resident and migrant caregivers, OAs, and employers [49]. The key is to ‘get along well’ [34]. Families face the need to care for OAs by bringing strangers into their homes [29]. As a result, the family members become employers and the home becomes a workplace where family members, OAs, and caregivers coexist [32]. According to our results, migrant caregivers reduce the burden on the primary caregiver [18,19,25,46]. Hiring a live-in caregiver allows for more person-centred care, relieving families of part of the caregiver burden [24], and can improve family relationships. However, it can also lead to greater financial vulnerability [20] and impose new demands on families [31,40,45], as a result of their role as employers. According to our results, differences in language, religion, culture, values, and expectations among OAs, families, and foreign caregivers influence the care experience [50]. The sense of uncertainty felt by the family members is exacerbated by negative preconceptions about immigration. In countries such as Spain, negative attitudes towards Moroccan and Latin American caregivers are still prevalent [51]. Given the lack of local caregivers for OAs, family members try to hire migrant caregivers who are caring, respectful, trained, and keen to learn [16,18,19]. Trust between the family members and migrant caregivers is key for both ensuring peace of mind for the family members and quality care for the OAs [29,33]. Although many family members maintain a relationship of trust with their domestic caregiver [19], others are wary, opting for vigilance and supervision [18,31]. While the lack of trust can be attributed to prejudice, cultural differences, language barriers and poor training [16,18,31,41,49,52], the urgency to hire caregivers lowers the expectations and demands of the family members. Living under the same roof places OAs, family members and migrant caregivers in an interdependent relationship [32]; employers often find themselves dealing with the migrant caregivers’ personal matters and administrative procedures or providing them with emotional support [31]. The family’s perception of the relationship with the migrant caregiver is usually positive [18,19], which improves the longer the caregiver is in the home [53]. However, while some family members consider the caregiver to be part of the family [19], others describe it simply as a working relationship. This contrasts with the caregivers’ perspective, as they express a desire to treat and be treated as part of the family [54]. They may even express fear of the caregiver leaving or of confronting them as it may have negative consequences on the OAs [18]. Any disagreements tend to be related to money, schedules, care or housework [31]. Many family members also feel supported by the migrant caregivers [16,41], which correlates with their level of education [16,53,55]. However, family problems with migrant caregivers are common [18,19,31,56], leading to a breakdown in trust and the caregiver leaving the job. Cultural differences pose an obstacle to forming bonds with migrant caregivers due to factors such as beliefs, customs, hygiene, clothing, diet or communication problems [53]. Overall, many families have limited resources to hire migrant caregivers for home-based care [31], and therefore require support from social and health services. It is important to strengthen social and financial support for families with OAs [18], regulate this labour market and implement plans for migrant domestic caregivers [44]. The lack of training for migrant live-in care providers is a significant concern for family members [16], and this is an area where nurses could play a role [49]. According to our findings, the caregivers’ peace of mind is crucial for the families, but it is not easy to achieve, as they must also care for their own families [54,57]. Anticipating caregiver burden and social support needs may be key to the management of frail OAs who are cared for by migrant caregivers [58]. Nevertheless, migrant caregivers usually integrate into the social milieu of the OAs and they participate in activities together. For the family members, the key lies in mutual respect for the traditions and beliefs of everyone involved in the care of the OAs [19].

### Limitations

Our meta-synthesis found a limited number of articles, small sample sizes, and focused exclusively on family members in six countries. The authors’ cultural background, and the fact that two of them had personal experiences as family members of OAs with migrant caregivers in their homes, may have influenced the interpretation of the findings. The cultural and social–healthcare service heterogeneity of the primary studies, together with the underrepresentation of male experiences and certain socioeconomic contexts among participants, may limit the study’s conclusions. Although this review draws on studies from a range of sociocultural contexts, restricting the search to English and Spanish may have introduced language bias, as relevant articles published in other languages may have been excluded. The restrictive nature of some search equations may have limited the number of studies identified. Nevertheless, although the search results addressed the research question, the transferability of the findings may be limited. The protocol and the review were not registered.

## 5. Conclusions

The results of our study highlight the difficulties, demands and challenges faced by family members when hiring migrant caregivers to care for OAs at home. While bringing a caregiver into the home can alleviate the burden on family caregivers, it can also become an additional source of stress and worry. Family members seek respectful and trained caregivers to whom they can delegate the care of the OAs. Hiring migrant caregivers involves bringing a stranger into the home, which can lead to feelings of doubt and mistrust. Over time, interdependence and trust develop between the family, OAs and migrant caregivers but family members feel they must remain vigilant nonetheless. Problems related to finances, work, beliefs, customs, diet, hygiene and social relationships are common and often lead to the caregiver leaving. Family members fear this departure and struggle to keep migrant caregivers with their elderly relatives. In such a case, they are forced to look for others from a similar culture or nationality, and the adaptation process starts all over again. Ensuring good coexistence and care requires reaching agreements between OAs, family members, and live-in migrant caregivers. The primary goal is the well-being of OAs, which involves overcoming cultural prejudices, learning together, improving caregiver training, and ensuring continuity of care.

## 6. Relevance to Clinical Practice

Family and community nursing could play a key role in training families and live-in migrant caregivers and implementing interventions aimed at basic medication management, assistance with basic activities of daily living, bedridden patient care, first aid, nutrition, and physical activity. Social workers could contribute to training family members on how to find migrant caregivers with legal residency status in the country, how to manage the hiring process, contract types, how to apply for financial assistance for employing families, and how to arrange for replacements to provide respite for families and caregivers. This training is applicable prior to hiring migrant caregivers through courses and practical seminars. It would be beneficial to schedule regular follow-up visits by community nurses and social workers, including periodic home visits, to assess problems and address emerging needs. Likewise, it would be beneficial to develop electronic applications that facilitate contact and communication between families, migrant caregivers, and social and healthcare professionals. Future research is needed on LTC at home provided by migrant caregivers to provide a better understanding of its impact on the quality of life of the OAs and their family members.

## Figures and Tables

**Table 1:** Strategies of search.

Databases	Search Strategy	Results
CINAHL	XB (family OR relatives) AND TI (qualitative) AND XB (migrant care)	27
PubMed	((((“migrant care worker” [Title/Abstract]) AND (“family” [Title])) OR (“relatives”[Title])) AND (“qualitative”[Title]))))	225
SCOPUS	TITLE-ABS-KEY (famil* OR relatives OR employer) AND TITLE-ABS-KEY (experiences OR perception) AND TITLE-ABS-KEY (migrant AND care AND worker) AND TITLE-ABS-KEY (qualitative AND research)	111
Web of Science	(((((((TS=(famil*)) OR TS=(relatives)) OR TS=(employer)) AND TS=(experiences)) OR TS=(perception)) AND TS=(migrant care worker)) AND TS=(qualitative research))	223
TOTAL		586

**Table 2:** Evaluation of the quality of the studies.

Article	1	2	3	4	5	6	7	8	9	10
Petry et al. (2016) [25]	✔	✔	✔	✔	✔	✔	↔	✔	✔	✔
Mehta & Leng, (2017) [44]	✔	✔	↔	✔	✔	↔	✔	✔	✔	✔
Morales-Gázquez et al. (2020) [19]	✔	✔	✔	✔	✔	✔	✔	✔	↔	✔
Munkejord et al. (2021) [36]	✔	✔	✔	✔	✔	✔	✔	✔	✔	✔
Yuan et al. (2022) [16]	✔	✔	✔	✔	✔	↔	↔	✔	✔	✔
Cohen-Mansfield & Golander (2023) [18]	✔	✔	✔	✔	✔	↔	↔	✔	✔	✔
Ness & Silan (2023) [45]	✔	✔	✔	✔	✔	✔	✔	✔	✔	✔
Hoens & Smetcoren (2023) [20]	✔	✔	✔	✔	✔	✔	↔	✔	↔	✔
Arieli & Halevi, 2024 [31]	✔	✔	✔	✔	✔	↔	↔	✔	✔	✔
Yen (2025) [46]	✔	✔	✔	✔	✔	✔	↔	✔	✔	✔
Ayalon et al. (2025) [47]	✔	✔	✔	✔	✔	✔	↔	✔	✔	✔

✔ Yes, ↔ Unclear, ✘ No. 1. Congruence of philosophical perspective/methodology 2. Congruence of methodology/objectives 3. Congruence of methodology/data collection 4. Congruence of methodology/data analysis 5. Congruence of methodology/interpretation of results 6. Cultural and theoretical context of the researcher. 7. Influence of the researcher on the research 8. Participants represented. 9. Research ethics committee approval. 10. Conclusions from data analysis/interpretation.

**Table 3:** Stages in the thematic synthesis process.

Stage	Description	Steps
Stage 1	Text coding	Recall review question Read/re-read findings of the studies Line-by-line inductive coding Review of codes in relation to the text
Stage 2	Development of descriptive themes	Search for similarities/differences between codes Inductive generation of new codes Write preliminary and final report
Stage 3	Development of analytical themes	Inductive analysis of sub-themes Individual/independent analysis Pooling and group review

**Table 4:** Characteristics of the chosen studies.

Author and Year	Country	Sample	Design	Data Collection	Data Analysis	Main Theme
Petry et al. (2016) [25]	Switzerland	15 family members	Grounded Theory	Semi-structured interviews	Grounded Theory techniques	A successful care system includes relationships and negotiated coexistence within the family network.
Mehta & Leng (2017) [44]	Singapore	30 family members	Descriptive qualitative study	In-depth interviews	Thematic analysis	Complexities when paid and unpaid family care is juxtaposed in the family household.
Morales-Gázquez et al. (2020) [19]	Spain	9 family members	Qualitative study with phenomenological approach	In-depth interviews and a focus group	Corbin & Strauss	Reasons for hiring a migrant caregiver and adaptation of family members during the process
Munkejord et al. (2021) [36]	Taiwan	10 family members	Qualitative interpretative and constructivist research	Semi-structured conversations	Braun & Clarke analysis	Interrelationships and collaboration between family members and caregivers
Yuan et al. (2022) [16]	Singapore	15 family members	Mixed-methods study	Semi-structured interviews	Braun & Clarke thematic analysis	Support and challenges for family members of people with dementia in hiring a migrant caregiver.
Cohen-Mansfield & Golander (2023) [18]	Israel	117 caregiver–family dyads	Mixed-methods study	One-on-one interviews	Braun & Clark thematic analysis, theory founded on Strauss & Corbin	Family members’ experiences of the relationship with migrant caregivers and development of interdependence.
Ness & Silan (2023) [45]	Taiwan	11 family employers	Qualitative interpretative and constructivist research	Narrative interviews	Narrative hermeneutic analysis	Understand of the transition from family carer to employer among indigenous families
Hoens & Smetcoren (2023) [20]	Belgium	8 family members	Descriptive qualitative study	In-depth interviews and a focus group	Braun & Clark thematic analysis	Experiences of hiring and living with a migrant caregiver.
Arieli & Halevi (2023) [31]	Israel	35 family caregivers	Descriptive qualitative phenomenological approach	Semi-structured in-depth interviews	Inductive content analysis	Experiences and emotional challenges of family employers of migrant caregivers
Yen (2025) [46]	Taiwan	4 family caregivers	Qualitative study	In-depth interviews	Thematic analysis	Motivations behind families’ decisions to hire migrant workers for home-based dementia care.
Ayalon et al. (2025) [47]	Israel	17 family members	Descriptive qualitative study	In-depth interviews	Braun & Clark thematic analysis	How people of different cultures and different care roles negotiate the understanding of dementia and its care within the caregiving context.

**Table 5:** Themes, sub-themes and units of meaning.

Theme	Sub-Theme	Units of Meaning
3.1. Not an easy decision.	3.1.1 Hiring a migrant domestic caregiver: when choice becomes a necessity	Deteriorating health, only child, working woman, difficulty adapting to a nursing home, lack of social assistance, need to reduce the workload of the informal caregiver
	3.1.2 Demands of the family members	Household chores, affection, respect, conditions, lack of training, initiative, keen to learn
	3.1.3 Becoming an employer	Financial problems, salary increase, caregiver needs, caregiver overload, mediation
3.2. A stranger at the heart of family life	3.2.1 Mixed feelings: between doubt and trust	Increased responsibilities, support, insecurity, supervision, financial control
	3.2.2 We are all interdependent: grateful but vigilant	Balancing personal life with caregiving, gratitude, caring for the caregiver, mutual help, part of the family, working relationship, care recipient
3.3. Two worlds that meet and need each other	3.3.1 A joint effort	Emotional support, physical support, training, household organisation
	3.3.2 When problems arise	Manipulation, deception, departure, financial gain, extortion
	3.3.3 Culture shock	Adaptation, language barrier, customs, respect, rejection
3.4. Improving the integration of live-in migrant caregivers into family life	3.4.1 Reaching consensus	Family consensus, consensus with migrant caregiver, negotiation
	3.4.2 Overcoming cultural prejudices	Lack of local caregivers, recommendations, prejudices
	3.4.3 Learning together	Language barriers, caring for the caregiver, training the caregiver, integration into family and social life
	3.4.4 Ensuring continuity of care	Building relationships, safety of OAs, family’s peace of mind
	3.4.5 The well-being of OAs	Staying at home, feeling cared for, joining the caregiver

## Data Availability

The data presented in this study are available on request from the corresponding author (data repository submission/inclusion process).

## Supplementary Materials

The following supporting information can be downloaded at: https://www.mdpi.com/article/10.3390/healthcare14040483/s1, Table S1: Synthesis of Qualitative Research Guidelines.
